# Not normal: a simulation study comparing effect sizes for skewed psychological data

**DOI:** 10.3389/fpsyg.2026.1771029

**Published:** 2026-04-21

**Authors:** Ambra Perugini, Giulia Calignano, Massimiliano Pastore

**Affiliations:** Department of Developmental and Social Psychology, University of Padua, Padua, Italy

**Keywords:** Cohen's d, data simulation, effect size, non-normal data, overlapping index

## Abstract

Effect sizes are widely used in psychological science, but with a focus solely on mean differences. This study compares three mean-based indices: Cohen's *d*, the Common Language Effect Size (CLES), the parametric overlap coefficient derived from d (η_*p*_), to the non-parametric Overlapping Index (η), which focuses on the entire distributional differences. Using skew-normal distributions to generate controlled scenarios of violations of normality and variance homogeneity, and symmetry, we systematically manipulated mean differences, variance ratios, skewness, and sample size to evaluate each index in terms of Relative Mean Bias, Normalized Root Mean Square Error, and 95% Coverage. Cohen's *d*, CLES, and η_*p*_ showed near-perfect descriptive correlations; however, their performance differed substantially. Cohen's *d* consistently exhibited low bias, high precision, and accurate coverage across all scenarios, whereas CLES and η_*p*_ showed substantial bias and low coverage, particularly under skewness and heteroscedasticity. The non-parametric index η remained unbiased under shape differences and variance heterogeneity but performed less reliably when the populations truly overlapped. The results indicated that effect-size indices derived from *d* are not interchangeable, and that high empirical correlations do not guarantee same precision. Cohen's *d* remains the most robust estimator of a location difference, whereas, η provides a more complete view of the entire distribution. Here, we argue that researchers should select effect sizes based on their statistical properties and propose a shift toward interpreting effects in light of the full distribution, rather than through mean-based conventions.

## 1 Introduction

Researchers often act as if a single number could capture the entire contrast between two empirical worlds. In psychological research, standardized effect size metrics are ubiquitous. As suggested by Lakens (2013), Cohen's *d* is “the most widely used effect size in psychological research” and is frequently used in meta-analyses aggregating group differences. As proposed by McGraw and Wong (1992) and Mastrich (2021), the Common Language Effect Size (CLES) is “an appealing index of effect size,” offering an intuitive interpretation in terms of probabilities of superiority. In their standard parametric form, both Cohen's *d* and CLES answer to the distance between means under assumptions of smooth, symmetric, and homoscedastic distributions. Under those conditions, variance does not rebel, and tails do not bend.

However, the distributions underlying psychological data rarely obey this ideal. Even in purely simulated scenarios, once asymmetry, variance heterogeneity, and modest or large mean shifts are introduced, the data no longer show a single clean contrast in location. Skewed distributions, heavy right tails, and unequal dispersions create conditions where the “same” effect, expressed as a standardized mean difference, may emerge from very different shapes. Metrics that compress the entire contrast into a standardized difference in means can then misrepresent how groups truly diverge: they may accurately reflect a central shift while remaining blind to the structure of the tails or to the degree of overlap between distributions.

These distributional concerns are particularly important in cognitive and clinical research. Reaction-time (RT) data in cognitive tasks are usually right-skewed and long-tailed, a pattern repeatedly observed in memory and attention paradigms (Osmon, 2018). Although effect sizes are often calculated from participant-level summaries of RT (such as mean or slope across trials), these summaries can still show significant asymmetry and heteroscedasticity, especially with limited trials, outliers, or large individual differences. When group variances differ, alternative standardized mean difference indices have been proposed (Morris, 2008)to capture a location shift, yet they may miss diagnostically meaningful differences that reside in the tails or in other shape features of the distributions. More generally, effect-size measures that explicitly incorporate distributional similarity can detect divergences that are not reducible to mean differences, including cases where group means are similar but the distributions differ in skewness or dispersion.

A similar motivation applies in clinical psychology, where symptom distributions are often non-normal and characterized by floor effects in non-clinical samples and heavy right tails in clinical populations(Haqiqatkhah et al., 2023; Rubio-Aparicio et al., 2018). Under these conditions, asymmetric dispersion can inflate pooled variance, potentially attenuating standardized mean differences and related *d*-based indices (Sun and Cheung, 2020). Taken together, these empirical settings encourage evaluating distribution-sensitive measures alongside traditional mean-based effect sizes, aiming to clarify when *d*-derived indices are sufficient and when distribution-level information offers additional insight into group differences separation (Hanel and Mehler, 2019).

These considerations motivate the present investigation, which evaluates classical mean-based indices alongside a distribution-sensitive alternative, the Overlapping Index. As defined by Pastore and Calcagnì (2019), the Overlapping Index (η) formalizes a distribution-sensitive perspective by quantifying how much two empirical density functions intersect. Unlike parametric overlap measures derived from Cohen's *d* (such as the Parametric Overlap, η_*p*_), η does not rely on normality or equal variances and can detect differences that are found in skewness, kurtosis, or tail behavior.

Rather than assuming that mean-based indices suffice in all psychological data scenarios, we systematically examined how *d*, CLES, η_*p*_, and the distribution-sensitive η behave under controlled violations of symmetry and variance homogeneity. By embedding these indices within a common simulation framework, we aimed to clarify when classical mean-based metrics remain adequate and when distribution-based overlap measures provide complementary or superior information.

The present study compares the aforementioned four metrics: Cohen's *d*, CLES, η_*p*_, and η. Cohen's *d* represents a standardized mean difference, expressing the distance between group means in units of pooled standard deviation. The Common Language Effect Size (CLES) can be expressed as a probability of superiority and, under normality, is a deterministic monotonic transformation of *d*. Likewise, the parametric overlap coefficient (η_*p*_) is mathematically derived from *d* under the assumption of normality and equal variances. Thus, *d*, CLES, and η_*p*_ encode essentially the same information in different scales. Conversely, the non-parametric overlapping index η estimates the shared area between empirical density functions and does not rely on parametric information. Unlike *d*-based indices, η is sensitive to differences in skewness, kurtosis, and tail structure.

From a theoretical perspective, the four indices considered here fall into two conceptual families. Cohen's *d* represents a standardized difference in means, and both the Common Language Effect Size (CLES) and the parametric overlap coefficient (η_*p*_) are deterministic transformations of *d* under normality. Consequently, when distributions are symmetric and homoscedastic, *d*, CLES, and η_*p*_ encode the same fundamental quantity: a location shift expressed on different scales. Their numerical values are therefore monotonically related and highly correlated by construction.

Conversely, the non-parametric overlapping index η estimates the shared area between the empirical density functions of the two groups. Instead of summarizing a shift in central tendency, it measures distributional similarity directly and remains sensitive to differences in skewness, kurtosis, and tail behavior structure. Thus, while *d*-based measures focus on standardized mean separation, η captures the overall configuration of the distributions.

Under approximate normality and homogeneity of variance, the three *d*-based indices should behave similarly both descriptively and inferentially. However, when distributions deviate from symmetry or exhibit heteroscedasticity, indices derived from *d* may remain mathematically linked yet differ in bias, precision, or coverage. Furthermore, the distribution-sensitive η may capture aspects of divergence not reflected in mean-based metrics.

The evaluation of the four indices will be conducted across a controlled set of non-normality scenarios generated from skew-normal distributions. By systematically varying mean differences, variance ratios, skewness, and sample sizes, and by computing all four indices on the same simulated datasets, we examined both how strongly they agree in practice and how effectively they recover the underlying population contrasts. We evaluated their behavior using Relative Mean Bias, Normalized Root Mean Squared Error, and Coverage, thereby assessing not only the magnitude each index reports but also its bias, stability, and inferential reliability under violations of normality and homoscedasticity. These results revealed that when classical, mean-based storytellers (*d*, CLES, η_*p*_) essentially retell the same narrative, and when the distribution-based η instead traces a distinct contour of difference, grounded in how much the two distributions truly overlap, rather than in how far apart their means are appear. In this sense, Cohen's *d*, CLES, η_*p*_, and η can be regarded as four different storytellers: some summarize only where the centers are located, while others focus on how far and how often the distributions diverge in their shapes.

Of note, the simulation-based approach is useful because it allows the evaluation of effect-size indices under fully controlled conditions, where the data-generating mechanisms are explicitly known and can be manipulated independently. As emphasized in the broader methodological literature on model assessment and inference (Akaike, 1998; Burnham and Anderson, 2002; Wagenmakers and Farrell, 2004), the simulation provides a principled way to examine how estimators behave when data generating conditions depart from mere differences in mean and variance is not homogeneous between groups. By varying mean differences, variance ratios, skewness, kurtosis, and sample sizes, simulations make it possible to quantify bias, precision, and robustness in ways that real datasets are necessarily limited, idiosyncratic, and often confounded, cannot support. This framework reveals how each metric performs across common distributional structures found in psychological data, showing where traditional indices remain reliable and where they do not. Essentially, the simulation acts as an empirical stress test, providing a clearer understanding of how Cohen's *d*, CLES, η_*p*_, and η behave across a range of realistic data-generating scenarios.

## 2 The simulation study

### 2.1 Data generation

The simulation generates data sets based on density distributions in different scenarios. In particular, we used the Skew-Normal distribution (Azzalini, 1985), which is defined in the following way: given ξ ∈ ℝ, ω ∈ ℝ^+^ and α ∈ ℝ, then for *y* ∈ ℝ we have


$$\begin{array}{l} \mathrm{SN}\left(y|\xi ,\omega ,\alpha \right)=\frac{1}{\omega \sqrt{2\pi }}\exp\left[-\frac{1}{2}{\left(\frac{y-\xi }{\omega }\right)}^{2}\right]\\ \;[1+\text{erf}\left(\alpha \left(\frac{y-\xi }{\omega \sqrt{2}}\right)\right)] \end{array}$$

in which


$$\begin{array}{l} \text{erf}\left(z\right)=\frac{2}{\sqrt{\pi }}\int_{0}^{z}e^{-t^{2}}dt \end{array}$$

is the error function.

ξ is the location parameter, ω is the scale parameter and α is related to the skewness of the distribution. The case in which ξ = 0, ω = 1 and α = 0 corresponds to the standard normal distribution. This distribution has been chosen to generate the data as it allows us to model both the distance between means (the effect size), symmetry, and variance. Consequently, it is optimal for our purpose, as it allows us to have control over the parameters of mean, variance, skewness, and kurtosis.

Mean and variance of the Skew-Normal are respectively:


$$\begin{array}{l} \begin{array}{cc} \mu & =\xi +\omega \gamma \sqrt{2/\pi }\\ \sigma^{2} & =\omega^{2}\left[1-\frac{2\gamma^{2}}{\pi }\right] \end{array} \end{array}$$ (1)

in which $\gamma =\alpha /\sqrt{1+\alpha^{2}}$.

Based on Equation 1, we can determine the values to assign to the parameters ξ and ω as functions of μ and σ with:


$$\begin{array}{l} \begin{array}{cc} \xi & =\mu -\omega \gamma \sqrt{2/\pi }\\ \omega & =\sqrt{\frac{\sigma^{2}}{1-\left(2\gamma^{2}\right)/\pi }} \end{array} \end{array}$$ (2)

### 2.2 Simulation design

The experiment consists of comparing two samples. We generated the first sample from SN(0,1,0), which corresponds to a Standard Normal distribution with mean 0 and variance 1. We generated the second sample from SN(ξ,ω,α), where the parameters are manipulated to produce specific differences in means (δ), standard deviations (σ), and skewness (α) between the two populations. The choice of generating the first sample always from a Standard Normal distribution and to vary the second sample is made to keep under control the number of scenarios.

Overall, the experimental design comprises eight scenarios. In each scenario, the first sample remains fixed as SN(0,1,0)≡N(0,1), while the second sample is defined by the following parameter settings:

- μ = 0, 2; mean of the second population. As the first sample always comes from a population with a mean of 0, this parameter—the mean of the population from which the second sample is drawn—also represents the true difference between the means. Consequently, in the following sections of the study, we refer to this parameter as δ;
- σ = 1, 5; standard deviation of the second population;
- α = 0, 10; asymmetry varies in the second group;
- *n* = 10, 50, 100, 300, 500, 1000; sample size, equal in the two samples.

**Figure 1:**
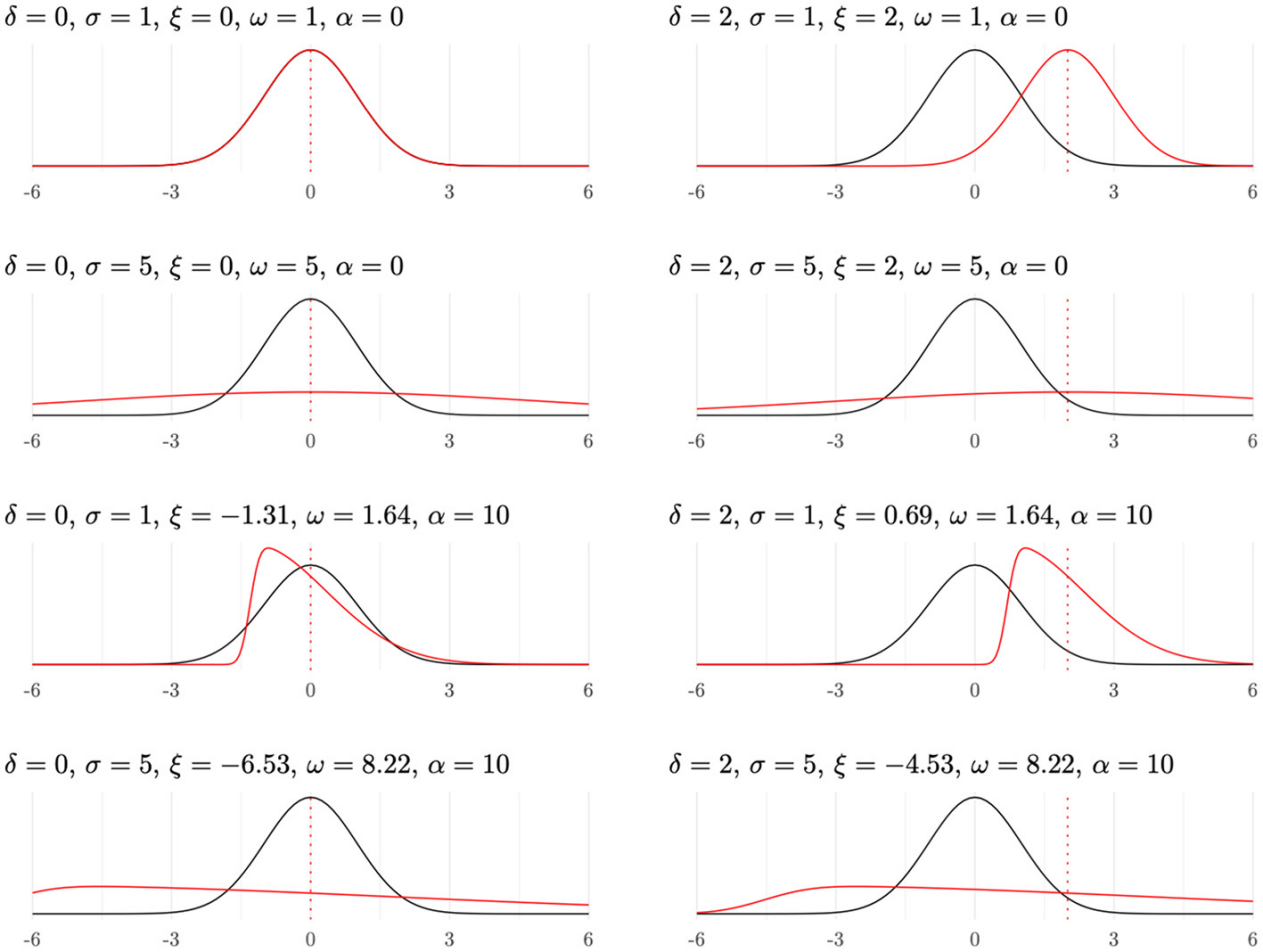
Representation of the simulation. In each panel, the black curves correspond to the first population SN(0,1,0)≡N(0,1), while the red curves correspond to the second population SN(ξ,ω,α).

Figure 1 depicts the experimental scenarios: the black curves correspond to the first population, consistently modeled as SN(0,1,0)≡N(0,1), whereas the red curves represent the second population SN(ξ,ω,α). The vertical dashed lines indicate the means of these second distributions, and also the mean difference, δ. Here δ is the mean difference and σ is the target SD of the second population; when α≠0, the skew-normal location and scale (ξ, ω) differ from the mean and standard deviation and are obtained by transforming (δ, σ, α) using Equation 2.

For each of the eight conditions, we generated 2000 pairs of independent samples and performed the analysis. On this generated data, for each combination δ × σ × α × *n*, we computed the following effect sizes:

- Cohen's *d*
- Common Language Effect Size (CLES).
- Two kinds of Overlapping Coefficient:
  1. Parametric (η_*p*_)
  2. Non-parametric (η).

### 2.3 Definition of effect sizes

#### 2.3.1 Cohen's d

Cohen's *d* is an effect size that standardizes differences in means between groups or conditions (Cohen, 1988). This index theoretically ranges from 0 to +∞ (for differences in one direction) or −∞ to 0 (for the opposite direction), but in practice, values rarely exceed a range of −3 to +3. A value of 0 indicates identical group means, whereas larger absolute values indicate greater centroid distance. In the simulated scenarios, it is appropriate to apply the between-groups formula:


$$\begin{array}{l} d=\frac{{\bar{X}}_{1}-{\bar{X}}_{2}}{s_{p}} \end{array}$$

where *s*_*p*_ is the pooled standard deviation defined as $s_{p}=\sqrt{\frac{\left(n_{1}-1\right)s_{1}^{2}+\left(n_{2}-1\right)s_{2}^{2}}{n_{1}+n_{2}-2}}$.

#### 2.3.2 Common Language Effect Size (CLES)

CLES represents the probability that a randomly selected observation from one group will exceed a randomly selected observation from the other group (McGraw and Wong, 1992). This index ranges from 0 to 1, with a value of 0.50 indicating complete overlap (no effect), values above 0.50 favoring one group, while those below 0.50 favor the other. Calculated from the data, the formula becomes:


$$\begin{array}{l} CL=\frac{N_{1}}{N_{1}+N_{0}} \end{array}$$

in which *N*_1_ indicates the number of ordered pairs in which the first observation is greater than the second, and *N*_0_ is the number of ordered pairs in which the first observation is smaller than the second.

It can also be calculated as a function of Cohen's *d*, Φ is the normal cumulative distribution function:


$$\begin{array}{l} CL=\Phi \left(|d|/\sqrt{2}\right) \end{array}$$

#### 2.3.3 Parametric overlapping

The concept of overlap refers to the shared area between two or more probability density functions. It offers an intuitive way to measure the level of similarity or difference between samples or populations characterized by their distributions. When two populations (or samples) exhibit a high degree of overlap in their distribution functions, it suggests a strong similarity between them. Conversely, minimal overlap indicates substantial differences.

The parametric version of the Overlapping coefficient can be derived from Cohen's *d* as follows:


$$\begin{array}{l} \eta_{p}=2\Phi \left(-|d|/2\right) \end{array}$$

where Φ is the normal cumulative distribution function.

#### 2.3.4 Non-parametric overlapping

The non-parametric version (η) is computed using a kernel approach and serves as a distribution-free approximation of η_*p*_. The Overlapping Index defines the area intersected by two or more empirical density functions (Pastore and Calcagnì, 2019). This index varies from zero (complete disjointment) to one (complete overlap) and can be considered an index of similarity between groups/conditions.

Assuming two probability density functions *f*_*A*_(*x*) and *f*_*B*_(*x*), the Overlapping Index η:ℝ^*n*^×ℝ^*n*^ → [0, 1] is formally defined in the following way:


$$\begin{array}{l} \eta \left(A,B\right)=\int_{{\mathbb{R}}^{n}}\min\left\{f_{A}\left(x\right),f_{B}\left(x\right)\right\}dx \end{array}$$

Where in the discrete case, the integer can be replaced by a summation. η(*A, B*) is normalized to one, and when the distributions of A and B do not have points in common, meaning that *f*_*A*_(*x*) and *f*_*B*_(*x*) are disjoint, η(*A, B*) = 0.

The computation of the index is implemented in the overlapping R package (Pastore et al., 2024).

### 2.4 Performance indexes for comparison

In order to compare effect size estimators' performance across conditions, we selected performance indices that are scale-free, that is, not affected by the magnitude or range of the underlying effect size. This choice is important because Cohen's *d* is unbounded and can take any real value, whereas the other indexes of interest are bounded between 0 and 1. As a result, performance measures defined on the raw scale would not be directly comparable across scenarios.

Conversely, the non-parametric overlap index targets the distributional overlap $\eta_{j}=\int_{{\mathbb{R}}^{n}}\min\left[f_{1}\left(x\right),f_{2}\left(x\right)\right]dx$ where *f*_1_ is the N(0,1) density and *f*_2_ is the skew-normal density under condition *j*. Therefore, performance indices (RMB, NRMSE, Coverage)quantify how well each estimator recovers its own θ_*j*_ across Monte Carlo replications.

Consequently, we used the following performance indices:

- Relative mean bias (RMB) captures the average proportional deviation between an estimator and its population value across Monte Carlo replications. It is defined as
$$RMB=\left(1/B\right)\underset{b}{\sum }\left[\left({\hat{\theta }}_{bj}-\theta_{j}\right)/\theta_{j}\right].$$
  where *B* is the number of replications, θ_*j*_ is the true parameter value (i.e., the target effect size) in condition *j* and ${\hat{\theta }}_{bj}$ is the estimated value in the *b* replication for condition *j*. Because this ratio expresses an error relative to the magnitude of the true parameter, it produces a scale-free measure of systematic distortion. Values close to zero indicate negligible bias, and values within the ±0.10 interval are typically interpreted as acceptable. Whenever the true value θ_*j*_ equals zero, a small constant (ϵ = 10^−6^) is substituted to prevent undefined divisions, ensuring numerical stability across replications.
- *Normalized root mean square error* (NRMSE) extends this scale-free approach to overall estimation error. It is simply derived from the Root Mean Squared Error (RMSE) index, dividing it by the empirical range of observed estimates,
NRMSE=RMSE/(max(obs)-min(obs)),
  where max(obs)−min(obs) is the empirical range of the *B* estimates ${\left\{{\hat{\theta }}_{bj}\right\}}_{b=1}^{B}$ under condition *j* and RMSE is:
$$\text{RMSE}=\sqrt{\frac{1}{B}\overset{B}{\underset{b=1}{\sum }}{\left({\hat{\theta }}_{bj}-\theta_{j}\right)}^{2}},$$
  where *B* is the number of replications, ${\hat{\theta }}_{bj}$ is the estimate in replication *b* under condition *j*, and θ_*j*_ is the corresponding true value of the effect size. As with RMB, lower values indicate better performance.
- Coverage evaluates whether the inferential procedure is properly calibrated. For each condition *j* and each replicate, we computed a 95% confidence interval (CI) for the target effect size parameter θ_*j*_ and recorded whether it contains the corresponding true value. Empirical coverage is then the proportion of replicates for which θ_*j*_∈CI. In our implementation, 95% CIs for Cohen's *d* are obtained using the non-centrality parameter method. For the non-parametric overlap η, we used a percentile bootstrap CI, taking the 2.5^*th*^ and 97.5^*th*^ percentiles of the bootstrap distribution. For CLES and the parametric overlap, both defined as deterministic transformations of *d*, confidence intervals are obtained by applying the respective transformation to the lower and upper bounds of the 95% CI for *d*. Specifically, with [*d*_low_, *d*_high_] denoting the CI for *d*, the CI for CLES, defined $\text{CLES}=\Phi \left(|d|/\sqrt{2}\right)$, is obtained by evaluating this expression at *d*_low_ and *d*_high_. Likewise, for the parametric overlap η_*p*_ = 2Φ (−|*d*|/2), the interval is obtained by evaluating the same function at the CI bounds of *d* (with endpoints ordered appropriately due to monotonicity).

Notably, other traditional indices such as Absolute Mean Bias, Mean Squared Error and RMSE are less suitable in this context because they remain scale-dependent. The relative and normalized metrics above resolve this limitation by expressing accuracy on a common proportional scale.

## 3 Results

### 3.1 Descriptive statistics

**Figure 2:**
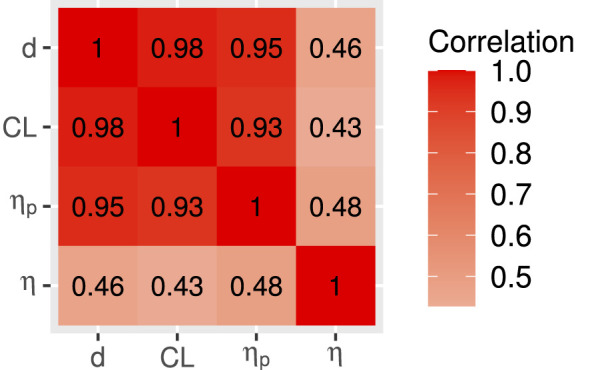
Pairwise Pearson's correlation between indexes. *d*, Cohen's *d*; CL, Common Language Effect Size; η_*p*_, parametric overlap; η, non-parametric overlap.

Figure 2 presents the pairwise Pearson's correlations among the four effect size indices considered in the study (Cohen's *d*, CLES, η_*p*_, and η) across all 48 simulated conditions.

Figure 2 has a very clear pattern, with Cohen's *d*, CLES and η_*p*_ showing very high correlations between each other, exceeding 0.90. Conversely, η has only moderate correlation with those three indexes, ranging from 0.43 to 0.58, suggesting that η reflects a more distinct component of the data. Taken together, these findings indicate that Cohen's *d*, CLES and η_*p*_ capture highly overlapping information about group differences, whereas η provides a partially independent characterization of the effect, consistent with the nature of the effect size that accounts for the shape of the distribution.

### 3.2 Performance indexes

To evaluate the performance of the four effect sizes, we examined their Relative Mean Bias (RMB), Normalized Root Mean Squared Error (NRMSE), and Coverage across all simulated scenarios.

#### 3.2.1 Relative Mean Bias (RMB)

**Figure 3:**
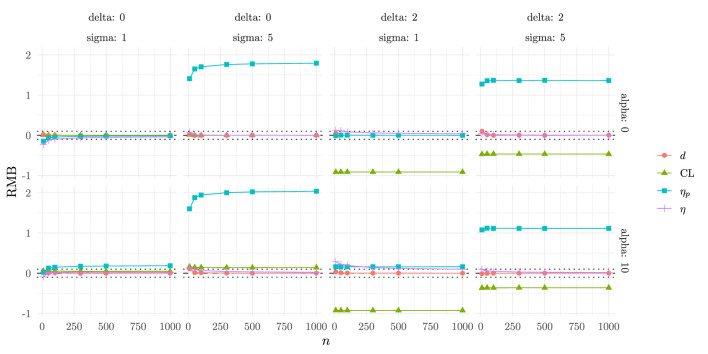
Relative Mean Bias as a function of the experimental conditions.

Figure 3 shows the RMB for each index as a function of sample size and under different experimental conditions. Overall, η and Cohen's *d* display the lowest bias, with values consistently close to zero across all conditions. CLES also exhibits relatively low bias across scenarios with equal means, but shows biased estimates when there is difference in means (δ = 2). Conversely, η_*p*_ shows unbiased estimates under same variance (σ = 1) and biased estimates when variance is non-homogeneous between the two groups (σ = 5).

#### 3.2.2 Normalized root mean square Error

**Figure 4:**
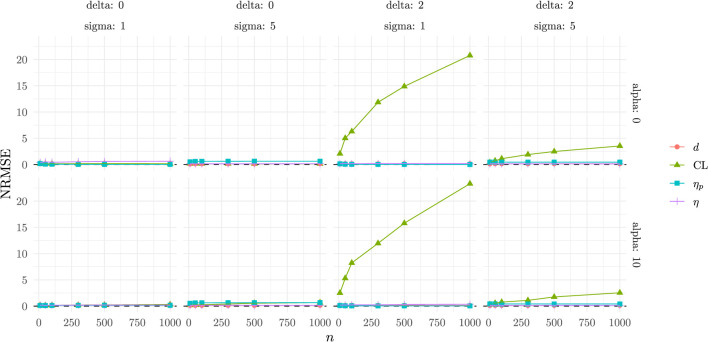
Normalized relative mean square error as a function of the experimental conditions.

The NRMSE results (Figure 4) show that CLES displays much larger NRMSE, dominating the scale of the plot and confirming its poorer performance in terms of stability and precision when there are differences in means between the two groups.

**Figure 5:**
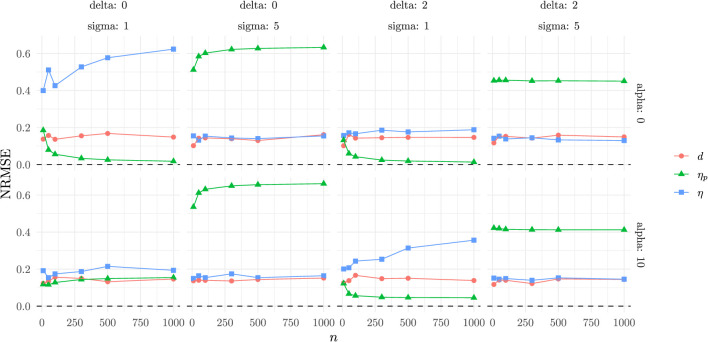
Normalized relative mean square error (without CLES) as a function of the experimental conditions.

For this reason, we included a second plot (Figure 5) in which CLES is excluded, as its performance is considerably worse than that of the other indexes.

In this comparison, Cohen's *d* emerges as the most stable estimator, with consistently lower NRMSE across all conditions. η_*p*_, shows high bias when the homogeneity of variance is not respected, but performs well under the same variance conditions. η performs at the level of Cohen's *d* in all conditions but one, when the two groups come from the same empirical distribution, reflecting the bounded nature of the index.

#### 3.2.3 Coverage

**Figure 6:**
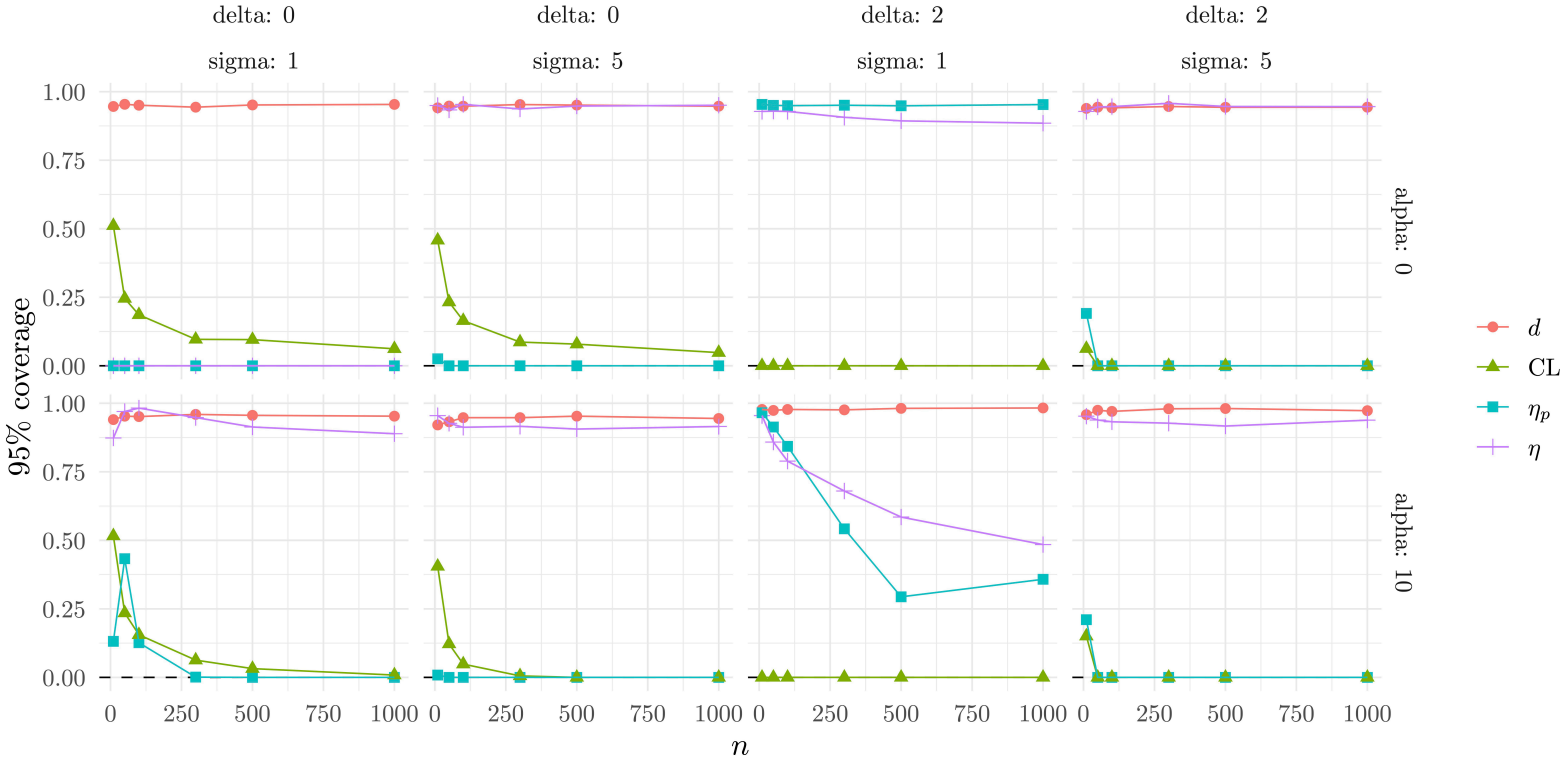
Coverage 95% as a function of the experimental conditions.

The 95% Coverage results (Figure 6) show how Cohen's *d* once more has good coverage in all scenarios, with η_*p*_ and CLES has the worst coverage across scenarios and η performing well in all scenarios, except in the one with δ = 0, σ = 1 and α = 0 and the one with δ = 2, σ = 1 and α = 10.

## 4 Discussion

The simulation results demonstrated that the four effect-size indices that is, Cohen's *d*, the Common Language Effect Size (CLES), the parametric overlap derived from *d* (η_*p*_), and the distribution-free Overlapping Index η do not constitute interchangeable descriptions of group or condition differences when distributions deviate from normality. Across all manipulated conditions (mean differences, variance ratios, skewness, and sample sizes), the indices separate into two functional families: mean-based location estimators (*d*, CLES, η_*p*_) and a distribution-sensitive overlap estimator (η). This separation reflects that the indices derived from *d* inherit all of its parametric limitations derived from its strict focus on means, as suggested by Peng and Chen (2014). However, the performance metrics show that this conceptual grouping does not imply equivalent inferential properties: although the three *d*-based indices behave similarly descriptively, they differ substantially in bias, stability, and coverage.

Across the full simulation space, near-perfect correlations among *d*, CLES, and η_*p*_ (ρ > 0.93) confirm their shared theoretical base. Each assumes that the primary quantity of interest is a standardized shift in location, with dispersion treated as a nuisance or absorbed through pooled variance. CLES is mathematically linked to *d* under symmetry, and η_*p*_ is a deterministic transformation of *d* under normality, as corrected by Grice and Barrett (2014). Thus, under approximately symmetric or mildly skewed distributions, all three indices encode the same latent structure, a rank-ordered monotonic recording of the mean distance. Nevertheless, their performances differ: Cohen's *d* shows low bias across all conditions and high coverage, indicating that it can identify the true parameter, but CLES and η_*p*_ show biased estimates and low coverage in some conditions, demonstrating that a high empirical correlation with *d* does not guarantee accuracy or inferential validity.

Out of the group, η diverges systematically from classical indices [*r*∈[0.42, 0.47]], not focusing strictly on means but capturing a broader distributional difference. Performing at the level of Cohen's *d* for RMB, its weakest performance or RMSR emerges under strict normality, probably due to the nature of the index. The index varies from 0, complete disjointment, and 1, complete overlap, but two empirical populations will never overlap exactly, even with a high sample size. Again, when the two populations' parameters of mean and variance are identical (δ = 0, σ = 1), particularly when they are completely the same, η shows low coverage, reflecting sensitivity to kernel estimation when true overlap is maximal.

In practical application, η relies on kernel-based density estimation, which smooths fluctuations and mitigates outliers (Chen et al., 2021). Importantly, the overlapping package permits adjustment of the kernel function, introducing a controllable dimension of flexibility, what might be called a tailored robustness check, that allows η to adapt to diverse data structures.

At last, Cohen's *d* is confirmed to be a robust tool to assess groups/conditions differences based on means. However, η, rather than standardized mean differences, quantifies shared mass between empirical densities, thereby capturing distributional aspects ignored by *d*-based measures. Simulation evidence confirmed that η remains unbiased even under strong variance heterogeneity (ratios of 3:1 or 4:1), unlike η_*p*_ and CLES, whose estimates become distorted under such violations (Skidmore and Thompson, 2013).

As already noted, these distributional issues are not merely theoretical but arise frequently in substantive areas of psychological research. Therefore, the present results are highly relevant for cognitive psychology and neuropsychology, where reaction-time (RT) measures are commonly right-skewed and long-tailed (Osmon, 2018), and for clinical psychology, where symptom variables often exhibit floor effects in non-clinical samples and heavy right tails in clinical populations (Haqiqatkhah et al., 2023; Rubio-Aparicio et al., 2018). In these settings, Cohen's *d* remains a meaningful summary of mean separation. In our simulations, it continues to recover the location shift reliably across conditions. However, the same results also highlight that mean-based indices alone may provide an incomplete description of group differences when distributions diverge in shape or dispersion. In particular, η summarizes distributional similarity directly and can therefore capture differences that are not reducible to a standardized mean contrast, including cases in which group means are similar but the distributions differ in skewness, tail behavior, or heterogeneity of variance. The complementary use of η becomes practically important when asymmetric dispersion increases pooled variance, a mechanism that can attenuate standardized mean differences and related *d*-based indices under non-normality and heteroscedasticity (Sun and Cheung, 2020). In such cases, reporting both a mean-based index (e.g., *d* or a related transformation) and η provides a balanced inferential summary: *d* anchors interpretation to location shifts that remain central for many research questions, whereas η adds information about the extent of distributional overlap that is directly tied to differences in the overall configuration of the data (Hanel and Mehler, 2019).

More practically, we recommend that researchers follow a simple set of steps when choosing between mean-based and overlap-based effect sizes. First, visualize the data (i.e., density/violin plots) and compute basic descriptive statistics (mean, median, variance, skewness, and kurtosis). Second, use this information to decide how to summarize group differences. When the distributions are clearly non-normal or differ in shape (e.g., skewness, tail behavior) and a mean-based index would miss substantively relevant dissimilarities, reporting the overlap index η provides a distribution-sensitive summary. When the research question still centers on mean separation (or comparability with prior work), reporting Cohen's *d* together with η can offer a clearer picture by combining a location-based effect size with a distribution-based measure of similarity. Finally, when distributions are approximately symmetric, variances are comparable, and the scientific focus is a location shift, Cohen's *d* alone may be sufficient. In all cases, we recommend reporting uncertainty (95% CIs), using standard analytic intervals for *d* and bootstrap-based intervals for η.

Here, η enters not as a rival, but as a companion. Where *d* fixes its gaze upon the center, η listens to the contour to the way distributions overlap, diverge, and rejoin across the span of shape variation (Hedges and Olkin, 2016). In doing so, it restores something that *d* leaves silent: the shape of difference. Their relationship is not one of substitution but of complementarity as two instruments, distinct in tone yet tuned to the same melody.

Thus, the lesson is not that one index should prevail, but that both deserve to speak. *d* translates the difference into the language of averages; η translates it into that of a broader concept of similarity between distributions. Furthermore, somewhere between those two dialects between what is measured and what is experienced lies the truer inference, the quiet understanding that every distribution in psychology, like every life, bends a little away from symmetry.

## 5 Conclusion

Effect sizes should not be treated as interchangeable. When the scientific question concerns a simple shift in location and indeed, no other significant difference in other parameters persists, *d* represents an adequate and robust index, with very little bias and high coverage of the true parameter. However, when such condition is not respected, and the question concerns distributional separation, like tail behavior, heterogeneity, multimodality, or skew-driven divergence η provides the useful and complementary description. An index as η can capture differences that would otherwise be overlooked when merely focusing on means. With the support of an adequate data visualization process, those differences become even clearer to the researcher and allow for a more tailored interpretation.

The simulation approach compares the effect size indexes employed here follows established methodological frameworks advocating controlled parameters manipulation to evaluate estimator performance (Zhao et al., 2020). The core emphasis of the present study is that no single effect-size metric suffices; robust interpretation depends on viewing several indices together and anchoring them to the shape and structure of the observed data. Simulations offer what empirical data cannot: a controlled environment for systematic stress testing. Simultaneously, it reflects the very distortions that shape real-world evidence, allowing us to understand how psychological effect-size indices hold or fail under those pressures.

## Data Availability

The original contributions presented in the study are included in the article/supplementary material, further inquiries can be directed to the corresponding author.
